# Endobronchial mucoepidermoid carcinoma in a pediatric patient: A case report

**DOI:** 10.1016/j.radcr.2022.03.043

**Published:** 2022-04-08

**Authors:** Michael Markovitz, Hector Monforte, Hester F. Shieh, Charles Jason Smithers, Jennifer Neville Kucera

**Affiliations:** a Department of Radiology, University of South Florida, Morsani College of Medicine, 2 Tampa General Circle, Tampa, FL STC 6102, USA; b Department of Pathology, Johns Hopkins All Children's Hospital, Saint Petersburg, FL, USA; c Department of Surgery, Johns Hopkins All Children's Hospital, Saint Petersburg, FL, USA; d Department of Radiology, Johns Hopkins All Children's Hospital, Saint Petersburg, FL, USA

**Keywords:** Pediatric, Pediatric radiology, Endobronchial tumor, Mucoepidermoid carcinoma

## Abstract

Mucoepidermoid carcinoma (MEC) is an uncommon type of salivary gland tumor that can present as an endobronchial neoplasm, most commonly in the adult population. Neuroendocrine carcinoid tumors comprise the majority of bronchial neoplasms in the pediatric population and are nearly indistinguishable from MEC on imaging. We present a rare case of MEC in a 3-year-old presenting with recurrent symptoms of lower airway obstruction and discuss its typical associated symptoms and imaging features.

## Introduction

Primary lung cancer is extremely rare in children with a differential that differs significantly from adults. One of the most common subtypes, bronchial tumors, consists of 3 histopathologically distinct diagnoses—mucoepidermoid carcinoma (MEC) or tumor, carcinoid tumor, and adenoid cystic carcinoma [1,2]. MEC is the most common type of salivary gland-type tumor of the lung, which accounts for 0.1% to 0.2% of all lung carcinomas [3,4]. These can be low or high-grade, with low-grade lesions more commonly occurring in younger patients [5]. MEC have an excellent prognosis relative to adenocarcinoma and squamous cell carcinoma, can occur at any age with a median of 40 years, and are not correlated with smoking [6]. Symptoms are nonspecific and range from cough, hemoptysis, or recurrent fever or pneumonia based on tumor location, but up to one-third of patients may be asymptomatic [6].

MEC tend to occur in relation to the tracheobronchial tree with a predilection for lobar bronchi over the trachea or mainstem bronchi [6]. Grossly they are exophytic tan-grey, yellow, or pink highly vascularized masses with a smooth mucosal surface. Pathologically they contain clear, squamoid, or transitional polygonal cells with interspersed mucus-secreting cells [3,^4^,6].

On imaging MEC appears as a well-circumscribed, smooth but nonspherical mildly enhancing intraluminal mass, often with associated bronchiectasis, mucous impaction, and distal atelectasis. Punctate calcification may be present in up to half of cases. Metastasis to regional lymph nodes is extremely rare [6].

## Case report

**Fig. 1:**
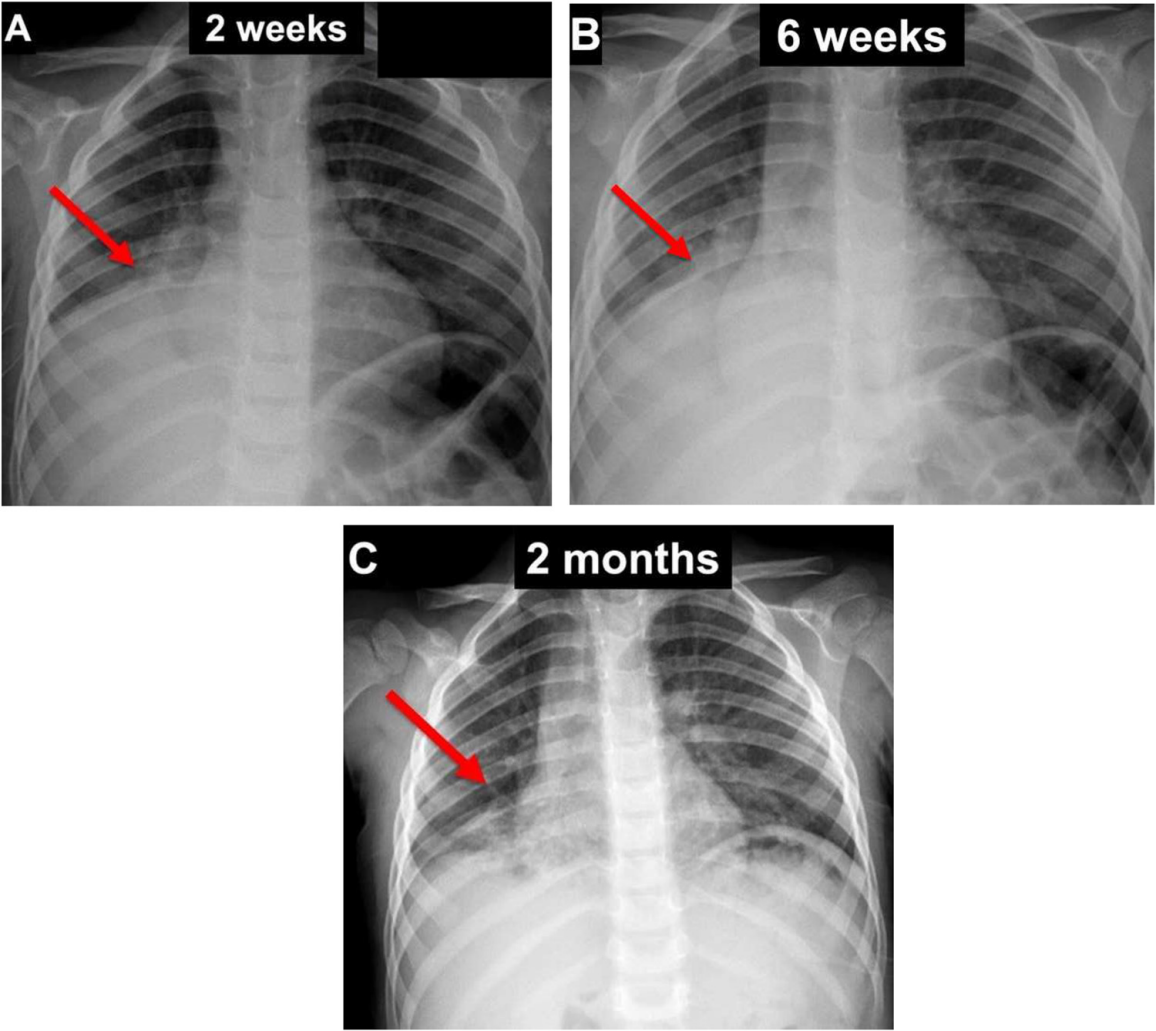
Sequential frontal chest radiographs approximately (A) 2 weeks, (B) 6 weeks, and (C) 2 months from initial symptoms show a relatively unchanged right middle and lower lobe consolidation over the course of 2 months.

A 3-year-old otherwise healthy male presented with cough, fever, and vomiting. He was diagnosed with upper respiratory infection and treated with a course of antibiotics. He returned 2 weeks later with recurrent symptoms when chest radiograph demonstrated right middle and lower lobe pneumonia (Fig. 1A). Symptoms mildly improved initially on a different antibiotic, but he presented to the emergency room 1 month later (6 weeks from initial presentation) with the same symptoms and unchanged chest x-ray (Fig. 1B). A stronger antibiotic regimen was trialed without improvement in symptoms or imaging findings 2 months from initial presentation (Fig. 1C).

**Fig. 2:**
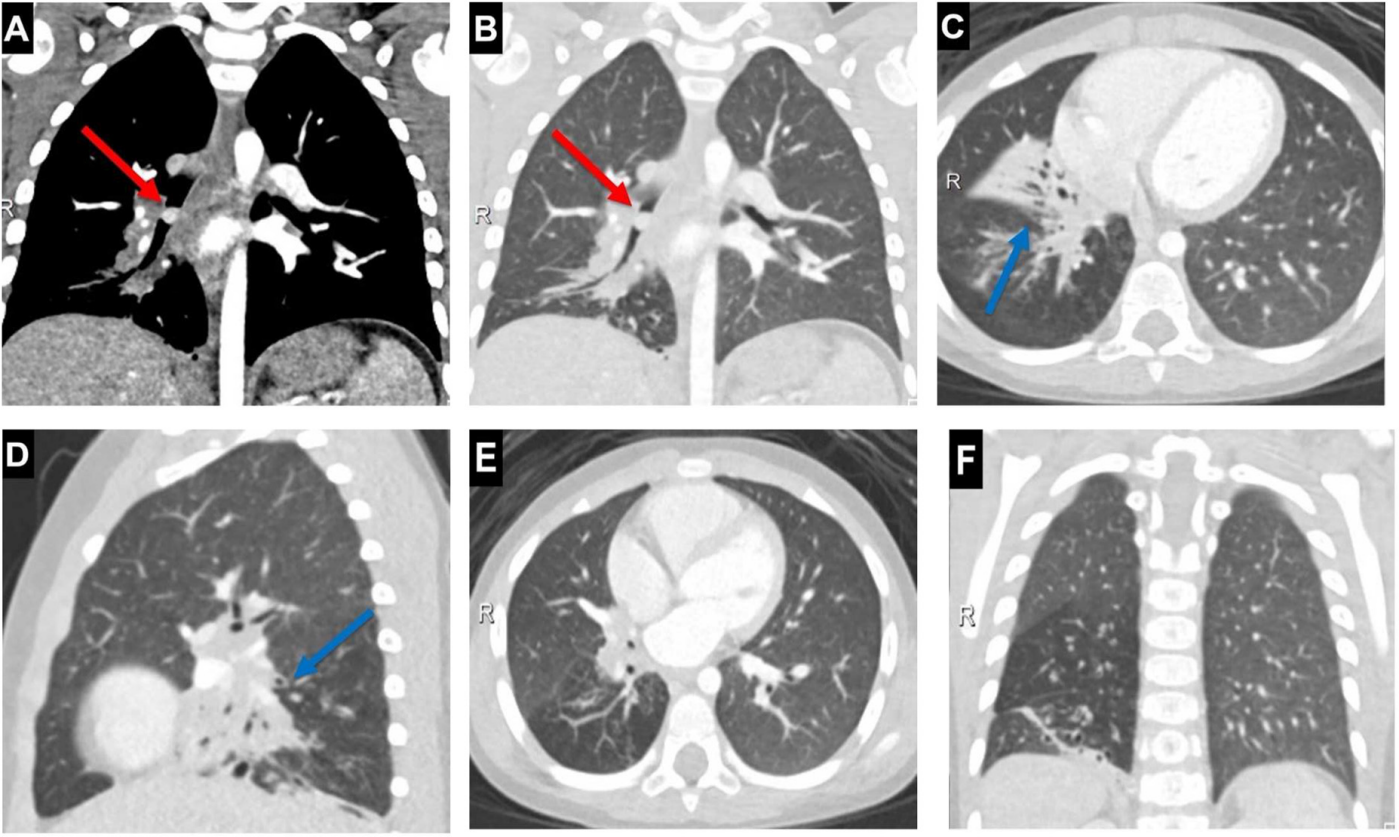
Axial, coronal, and sagittal CT images with lung and soft tissue windows demonstrate (A, B) a 9 mm mildly enhancing, exophytic endobronchial mass arising from the right bronchus intermedius (red arrows). (C, D) Postobstructive consolidation and bronchiectasis (blue arrows) are present along with (E, F) associated air trapping evidenced by scattered areas of hyperlucent lung parenchyma (green arrows) (Color version of figure is available online).

**Fig. 3:**
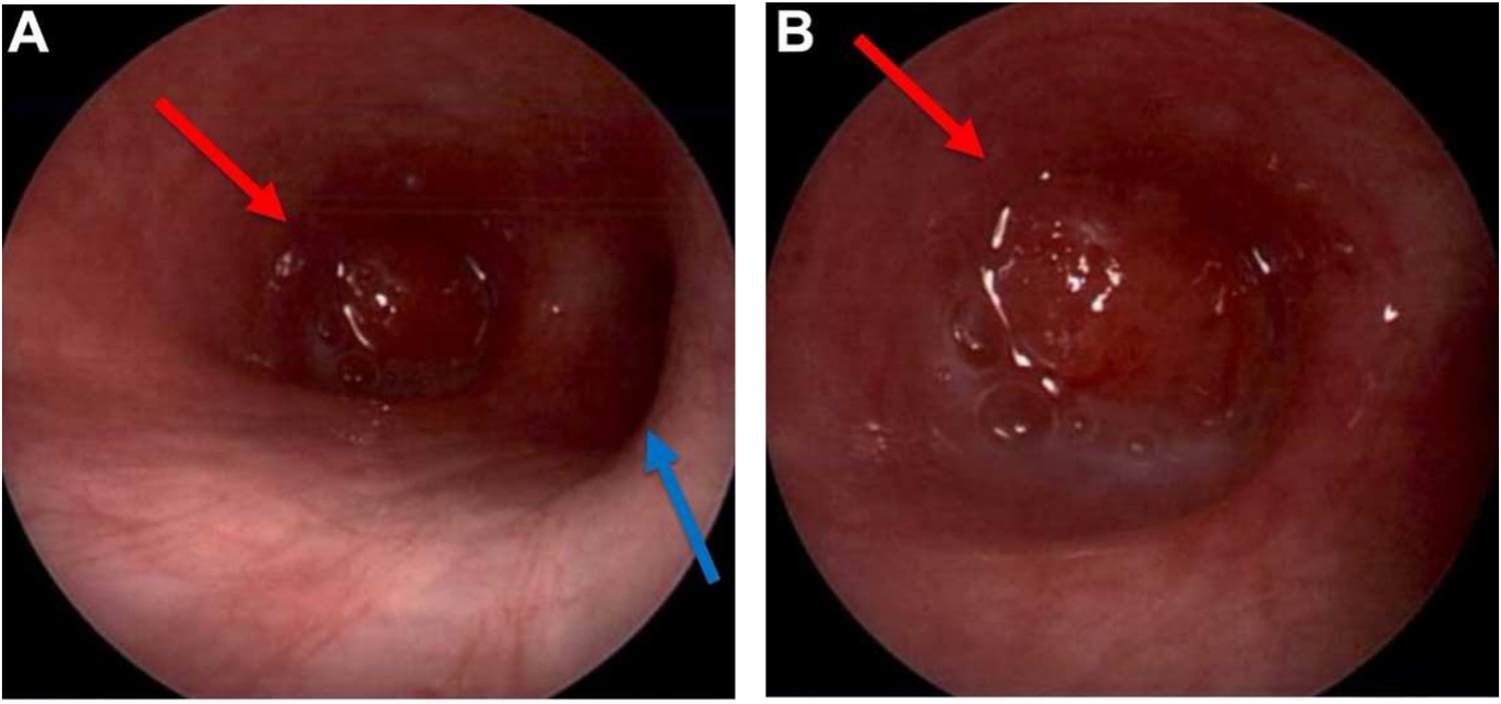
Bronchoscopic images in the (A) right mainstem bronchus and (B) bronchus intermedius demonstrate an obstructive mass (red arrow) just distal to the patent right upper lobe bronchus (blue arrow) (Color version of figure is available online).

**Fig. 4:**
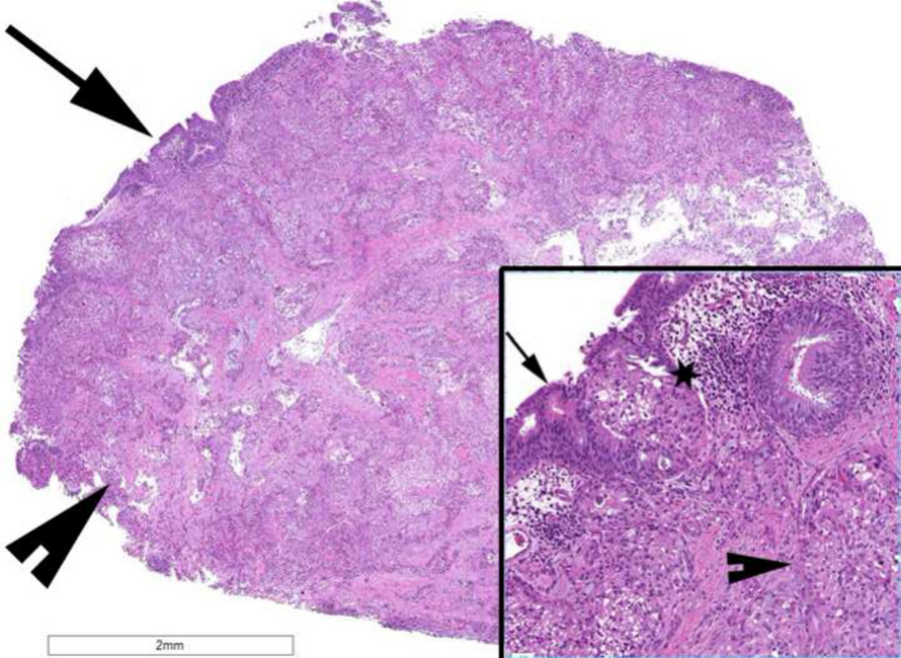
H&E-stained section at 10× magnification of whole 9 mm infiltrative mucosal tumor (arrow) composed of islands and cords of intermediate cells and mucin producing cells, expanding the underlying lamina propria (arrowhead). Tumor extended to the mucosal and deep margins. No bronchial muscle or cartilage was present. At 40× magnification superficial mucosal involvement is appreciated (*) with invasive tumor underneath.

The decision was made to further evaluate with computed tomography (CT), which showed a 9 mm mildly enhancing, endobronchial mass arising from the right bronchus intermedius. Associated right lower lobe consolidation, bronchiectasis, and air trapping were also present (Fig. 2). Based on the imaging features, the main differential considerations included carcinoid tumor along with less common endobronchial lesions, yet chronic foreign body could not be entirely excluded at this point. Bronchoscopy confirmed an endobronchial mass arising from the bronchus intermedius just distal to the takeoff of the right upper lobe bronchus, which was removed with no obvious residual mass (Fig. 3). The mass was extracted fairly easily, such that it gave the initial impression of a chronic foreign body; however, pathology revealed characteristic morphology of low-grade bronchial mucosal MEC infiltrating the mucosa and lamina propria (Fig. 4).

The case was discussed at multidisciplinary tumor conference with the plan for surgical resection of the affected portion of airway and the associated lobes of the lung. Other options were considered including observation with repeat bronchoscopy and/or imaging follow up, endoscopic bronchial mucosal resection or obliteration with close follow up, and bronchial sleeve resection sparing lung removal. Given his young age and the compensatory lung growth that occurs for patients younger than 5 to 6 years of age, the more aggressive surgical approach was taken to achieve the highest likelihood for long term cure. Furthermore, one would expect completely normal respiratory function following this resultant lung growth. He therefore underwent right thoracotomy with right middle and lower lobectomy, resection of the bronchus intermedius up to the right upper lobe takeoff under bronchoscopic guidance, and adjacent lymph node dissection. There were no obvious metastases; however, chronic scarring and inflammation of the affected lobes was severe. The proximal airway margin was free of tumor on frozen section. Resected right middle and lower lobes showed extensive chronic bronchitis and bronchiolitis with mucosal fibrous thickening throughout. No residual recognizable tumor or tumor biopsy field could be identified. Lymph nodes were devoid of any metastatic disease.

## Discussion

Some of the most common causes of lower airway obstruction in the pediatric population include asthma, pneumonia, bronchiolitis, laryngotracheo-bronchitis, congenital malformation, foreign body aspiration, and bronchial neoplasm [7,8]. When pediatric patients with recurrent respiratory symptoms fail to respond to conventional therapy, pathologic obstruction should be considered [9]. In this patient, the focal endobronchial lesion identified on CT greatly narrowed down this differential.

Foreign body aspiration was a primary diagnostic consideration in this patient given his age. Symptoms of foreign body aspiration range from nonspecific cough, fever, and chronic infection as in our patient to more serious bronchial rupture or bronchopulmonary fistula formation [10]. Most aspirated foreign bodies are radiolucent; however, one would typically expect to see associated hyperinflation which was not a predominant feature in our case.

Although bronchial neoplasms are less common than foreign body aspiration, carcinoid tumors make up the majority of these [1,^2^,10]. Endobronchial neuroendocrine carcinoid tumors and MEC are difficult to differentiate clinically as they present with similar symptoms and imaging features. On contrast-enhanced CT both appear as enhancing intraluminal masses with calcification present in less than half of cases. Carcinoid may enhance more than MEC but this is variable [10].

In most cases bronchoscopy with biopsy provide the fastest and most informative diagnostic confirmation. Adjunctive nuclear medicine studies may be performed to differentiate the two. A positive Ga-DOTATE scan would suggest typical carcinoid, whereby a negative result would point towards MEC [6]. Similarly, a positive radionuclide-tagged octreotide study can confirm the diagnosis of carcinoid tumor [10]. FDG PET can be performed in older patients with high-grade lesions, but is of little utility in low-grade lesions, such as this patient, which may only exhibit mild uptake [11]. None of these nuclear medicine studies were performed in this patient as the distinction was made by diagnostic pathology on biopsy. Additionally, these studies would have exposed the patient to additional radiation in this younger patient and likely would have been nondiagnostic given the small 9 mm lesion size.

Regardless of the imaging findings, bronchoscopy with biopsy is necessary for accurate diagnosis. Foreign body removal from the airway will generally resolve the respiratory infections associated with airway obstruction, and then time can be allowed for lung recovery. Some patients may still require lung resection for chronic infection or bronchiectasis. A treatment plan that includes complete tumor resection is required for all the above-mentioned tumors. The specific anatomy of the tumor will determine what type of operation is optimal. Other factors including patient age, medical comorbidities, and tumor grade/stage will also impact surgical planning. Endoscopic methods of resection or mucosal obliteration (eg, laser) are sometimes appropriate. Bronchial resections that spare lung removal can also be performed, especially for patients older than 5 to 6 years of age.

## Patient consent

The authors confirm that consent for publication has been obtained from the patient or his/her representatives.
